# F227. PSYCHOLOGICAL TRAUMA OCCURRING DURING ADOLESCENCE IS ASSOCIATED WITH AN INCREASED RISK OF GREATER WAIST CIRCUMFERENCE IN EARLY PSYCHOSIS PATIENTS INDEPENDENTLY OF MEDICATION

**DOI:** 10.1093/schbul/sby017.758

**Published:** 2018-04-01

**Authors:** Luis Alameda, Axel Levier, Philippe Golay, Mehdi Gholam-Rezaee, Frederik Vandenberghe, Aurélie Delacretaz, Anaïs Glatard, Céline Dubath, Kim Q Do, Chin B Eap, Philippe Conus

**Affiliations:** 1 Institute of Psychiatry, Psychology & Neuroscience, King’s College London; 2 Lausanne University Hospital, Hospital of Cery; 3 Lausanne University Hospital; 4 Centre Hospitalier Universitaire Vaudois (CHUV), University of Lausanne (UNIL)

## Abstract

**Background:**

The high prevalence of obesity in patients suffering from psychosis is a major concern as it dramatically increases the mortality rates of such patients in the long term. The mechanisms by which these patients develop overweight are poorly understood. It has been suggested that exposure to Childhood Trauma (CT) may play a role in the risk for obesity; however, whether this is the case for Early Psychosis (EP) patients and independently of the impact of medication has yet to be investigated. In addition, it is unknown whether the age at the time of exposure to CT can modulate the link between CT and obesity in EP patients.

**Methods:**

136 EP patients aged 18–35 were recruited from the Treatment and Early Intervention in Psychosis Program (TIPP-Lausanne). Body Mass Index (BMI), Weight Gain (WG) and Waist Circumference (WC) were measured and monitored prospectively after psychotropic prescription during a follow-up period of 1 year (patients were assessed at baseline, after 1, 2, 3, 6 months and 1 year of antipsychotic treatment). Patients were classified into Early-Trauma if they had faced at least one experience of abuse (physical, sexual, or emotional) or neglect (physical or emotional) before age 12, and Late-Trauma if the exposure had occurred between ages 12 and 16. Linear Mixed effect models with a random intercept were used to investigate the impact of Trauma (early or late) on the metabolic parameters longitudinally, Marko Chain Monte Carlo (MCMC) method was used to adjust these models with sufficiently large number of MCMC iterations. Models were adjusted for age, socioeconomic status, baseline BMI, medication intake prior to the first assessment and during the treatment phase, and by the diagnosis of depression.

**Results:**

Patients were more likely to have a diagnosis of Schizophrenia (61%; N=83), they had a mean age of 26 at the time of first assessment, and exposure to 1 or more forms of traumatic experiences before 16 years of age was present in 32% of the sample. No differences between groups were found at baseline in terms of BMI or WC. Late-Trauma patients, when compared to Non-Trauma patients showed greater WCs during the follow-up (p=0.012). No differences between Early or Late-Trauma patients and Non-Trauma patients were found in any of the other outcome measures during the follow up. Baseline BMI and treatment duration were significantly associated with the level of BMI and WC during the follow up. None of the other potential confounding factors were significantly associated with the outcome measures during the follow up.

**Discussion:**

Exposition to trauma during adolescence in EP patients is associated with a higher risk of greater WC during the early phase of the disease, independently of the medication intake, depression and other confounding factors. Specific preventive measures should be addressed in these patients in order to reduce the risk of obesity. Depending on the timing of traumatic exposure, different developmental mechanisms may underlie this differential possible impact on WC. Further studies on interactions between central consequences of traumatism and metabolic syndrome are warranted.

